# Supplementary material for: A validation of machine learning-based risk scores in the prehospital setting
Source: PLoS One. 2019 Dec 13;14(12):e0226518. doi: 10.1371/journal.pone.0226518 (PMC6910679; doi:10.1371/journal.pone.0226518)
Supplement: S1 Analysis — Provides results from a Precision/recall curve analysis as commonly reported in the machine learning literature, presented in the same manner as Fig 1 and Table 2 in the main analysis. (DOCX) [file pone.0226518.s001.docx]

#
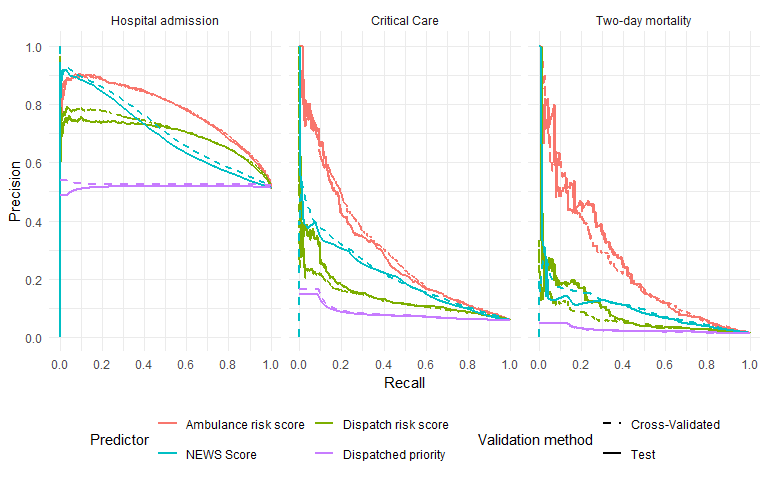
S1 Analysis - Precision/Recall analysis

Fig 1 Equivalent

Table 3 Equivalent

|  | | Area under Precision/Recall curve (95% CI) | | | |
| --- | --- | --- | --- | --- | --- |
| Validation method | Outcome | Dispatched priority | NEWS Score | Dispatch risk score | Ambulance risk score |
| Test | Hospital admission | 0.52 (0.51-0.52) | 0.70 (0.69-0.71) | 0.69 (0.68-0.70) | 0.78 (0.77-0.79) |
|  | Critical Care | 0.08 (0.07-0.09) | 0.20 (0.18-0.23) | 0.15 (0.13-0.17) | 0.29 (0.26-0.32) |
|  | Two-day mortality | 0.02 (0.02-0.03) | 0.10 (0.07-0.13) | 0.09 (0.06-0.13) | 0.25 (0.18-0.32) |
| Cross-Validated | Hospital admission | 0.53 (0.52-0.53) | 0.72 (0.71-0.72) | 0.71 (0.70-0.72) | 0.79 (0.78-0.79) |
|  | Critical Care | 0.08 (0.08-0.09) | 0.21 (0.19-0.23) | 0.13 (0.12-0.14) | 0.30 (0.28-0.33) |
|  | Two-day mortality | 0.02 (0.02-0.03) | 0.10 (0.08-0.12) | 0.06 (0.04-0.07) | 0.24 (0.19-0.29) |
