# Supplementary material for: A validation of machine learning-based risk scores in the prehospital setting
Source: PLoS One. 2019 Dec 13;14(12):e0226518. doi: 10.1371/journal.pone.0226518 (PMC6910679; doi:10.1371/journal.pone.0226518)
Supplement: S2 Analysis — Investigates the sensitivity of our results to a broadening of the inclusion criteria used in the main analysis, we report all tables, figures and supplementary analyses presented here, but including patients left on scene by an ambulance or transported to a non-ED destination. (DOCX) [file pone.0226518.s002.docx]

## S2 Analysis. Broad inclusion criteria.

This sensitivity analysis presents results based on a broader cohort of patients than that presented in the main analysis. In this analysis, patients transported to non-ED destinations, and patients left on scene by the ambulance are included. Hospital records were captured for patients left on scene if they visited the hospital within 72 hours of contacting the ambulance service. Note that vital signs are more often missing among these patients, making the estimation of NEWS scores more unreliable, and assumptions of at-random missingness are thus more tenuous. This cohort of patients is also subject to additional sources of loss to follow-up (e.g. due to the use of healthcare facilities outside of the studied region, and due to contacts outside of the 72 hour window for which we receive hospital data).

## Table 1

|  | Training dataset (2016-2017) | | | Test dataset (2018) | | |
| --- | --- | --- | --- | --- | --- | --- |
|  | Excluded, N | Excluded, percent | Remaining, N | Excluded, N | Excluded, percent | Remaining, N |
| Original |  |  | 45045 |  |  | 23623 |
| No dispatch CDSS data | 2256 | 5.3 | 42789 | 827 | 3.6 | 22796 |
| Missing PIN | 2113 | 5.2 | 40676 | 1244 | 5.8 | 21552 |
| No ambulance journal | 1095 | 2.8 | 39581 | 466 | 2.2 | 21086 |
| Transport with no hospital journal | 3958 | 11.1 | 35623 | 1429 | 7.3 | 19657 |
| Missing > 2 vitals | 4881 | 15.9 | 30742 | 3070 | 18.5 | 16587 |
| Patient age < 18 | 1501 | 5.1 | 29241 | 784 | 5 | 15803 |
| Included in study | 17337 | 38.5 | 27708 | 8317 | 35.2 | 15306 |

##

## Table 2

|  | Priority | | | |  |
| --- | --- | --- | --- | --- | --- |
|  | 1A | 1B | 2A | 2B | Total |
| N | 1377 | 17252 | 19555 | 4830 | 43014 |
| Age, mean | 56.0 (54.8-57.3) | 64.2 (63.9-64.5) | 67.8 (67.5-68.1) | 67.6 (67.1-68.2) | 65.9 (65.7-66.2) |
| Female, percent | 46.7 (44.0-49.5) | 49.8 (49.1-50.5) | 53.6 (52.9-54.3) | 54.1 (52.7-55.5) | 51.9 (51.4-52.4) |
| Emergent transport , percent | 37.5 (35.1-40.0) | 24.4 (23.7-25.0) | 3.9 (3.7-4.2) | 2.0 (1.6-2.4) | 13.0 (12.7-13.3) |
| Ambulance intervention*,  percent | 86.3 (84.5-88.2) | 86.0 (85.5-86.6) | 69.4 (68.7-70.0) | 60.4 (59.0-61.8) | 75.6 (75.2-76.0) |
| Missing vitals, percent | 33.7 (31.2-36.1) | 26.0 (25.4-26.7) | 24.2 (23.6-24.9) | 23.7 (22.5-24.9) | 25.2 (24.8-25.6) |
| NEWS value, mean | 5.69 (5.49-5.91) | 3.67 (3.61-3.72) | 2.98 (2.93-3.02) | 2.39 (2.31-2.46) | 3.27 (3.24-3.31) |
| Prior contacts (30 days), mean | 0.21 (0.18-0.25) | 0.17 (0.16-0.18) | 0.17 (0.16-0.17) | 0.23 (0.22-0.25) | 0.18 (0.17-0.18) |
| Intensive Care Unit, percent | 9.8 (8.2-11.5) | 3.2 (3.0-3.5) | 1.5 (1.4-1.7) | 1.4 (1.1-1.7) | 2.5 (2.3-2.6) |
| In-hospital death, percent | 8.6 (7.0-10.0) | 3.9 (3.5-4.1) | 3.7 (3.4-3.9) | 4.0 (3.5-4.6) | 3.9 (3.7-4.1) |
| Critical care, percent | 15.1 (13.1-17.0) | 6.1 (5.8-6.5) | 4.5 (4.2-4.8) | 4.4 (3.9-5.1) | 5.5 (5.2-5.7) |
| Admitted, percent | 50.2 (47.6-52.9) | 50.8 (50.0-51.6) | 50.4 (49.8-51.1) | 47.6 (46.1-49.0) | 50.2 (49.8-50.7) |
| 2-day mortality, percent | 4.6 (3.6-5.7) | 1.5 (1.3-1.7) | 0.8 (0.7-0.9) | 0.8 (0.5-1.0) | 1.2 (1.1-1.3) |

## Figure 1


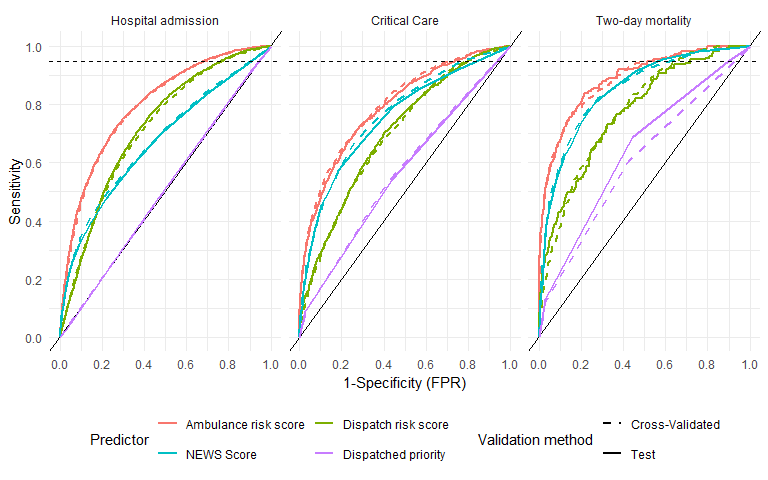


## Table 3

|  | | Concordance index (95% CI) | | | |
| --- | --- | --- | --- | --- | --- |
| Validation method | Outcome | Dispatched priority | NEWS Score | Dispatch risk score | Ambulance risk score |
| Test | Hospital admission | 0.51 (0.50-0.52) | 0.67 (0.66-0.68) | 0.72 (0.71-0.73) | 0.80 (0.79-0.80) |
|  | Critical Care | 0.56 (0.54-0.58) | 0.75 (0.73-0.77) | 0.70 (0.68-0.72) | 0.79 (0.77-0.81) |
|  | Two-day mortality | 0.64 (0.60-0.68) | 0.84 (0.81-0.87) | 0.77 (0.74-0.80) | 0.89 (0.86-0.91) |
| Cross-Validated | Hospital admission | 0.51 (0.50-0.51) | 0.68 (0.67-0.68) | 0.71 (0.71-0.72) | 0.79 (0.79-0.80) |
|  | Critical Care | 0.57 (0.56-0.58) | 0.77 (0.75-0.78) | 0.70 (0.68-0.71) | 0.80 (0.79-0.81) |
|  | Two-day mortality | 0.61 (0.58-0.63) | 0.85 (0.83-0.87) | 0.77 (0.75-0.80) | 0.88 (0.86-0.90) |

## Table 4

| Type | Priority | N | Emergent transport , percent | Ambulance intervention*,  percent | NEWS value, mean | Critical care, percent | Admitted, percent | 2-day mortality, percent |
| --- | --- | --- | --- | --- | --- | --- | --- | --- |
| Current | 1A | 507 | 34.5 (30.6-38.7) | 85.6 (82.4-88.6) | 5.51 (5.15-5.82) | 14.4 (11.2-17.6) | 47.5 (43.6-51.9) | 4.5 (2.8-6.5) |
|  | 1B | 6298 | 22.1 (21.0-23.1) | 84.9 (84.1-85.8) | 3.60 (3.51-3.69) | 6.0 (5.4-6.6) | 50.7 (49.5-51.9) | 1.5 (1.2-1.8) |
|  | 2A | 6940 | 3.1 (2.7-3.6) | 68.7 (67.6-69.7) | 2.83 (2.76-2.90) | 4.5 (4.0-4.9) | 50.1 (49.0-51.2) | 0.7 (0.5-0.9) |
|  | 2B | 1561 | 1.7 (1.0-2.3) | 58.6 (56.2-61.0) | 2.30 (2.17-2.43) | 4.5 (3.6-5.6) | 46.6 (43.8-49.1) | 0.6 (0.3-1.0) |
| By dispatch risk score | 1A | 507 | 36.1 (32.0-40.6) | 90.1 (87.6-92.9) | 7.14 (6.78-7.50) | 22.7 (18.9-26.2) | 74.2 (70.0-77.7) | 9.3 (6.9-11.8) |
|  | 1B | 6298 | 13.5 (12.7-14.4) | 75.4 (74.3-76.5) | 4.14 (4.04-4.23) | 7.7 (7.0-8.4) | 67.6 (66.4-68.8) | 1.5 (1.2-1.8) |
|  | 2A | 6940 | 9.9 (9.1-10.6) | 73.7 (72.6-74.8) | 2.38 (2.33-2.44) | 3.2 (2.8-3.6) | 39.9 (38.8-41.1) | 0.5 (0.3-0.6) |
|  | 2B | 1561 | 5.8 (4.6-7.0) | 73.3 (71.1-75.5) | 1.58 (1.49-1.66) | 0.9 (0.4-1.3) | 14.8 (13.0-16.5) | 0.1 (0.0-0.2) |

##

##
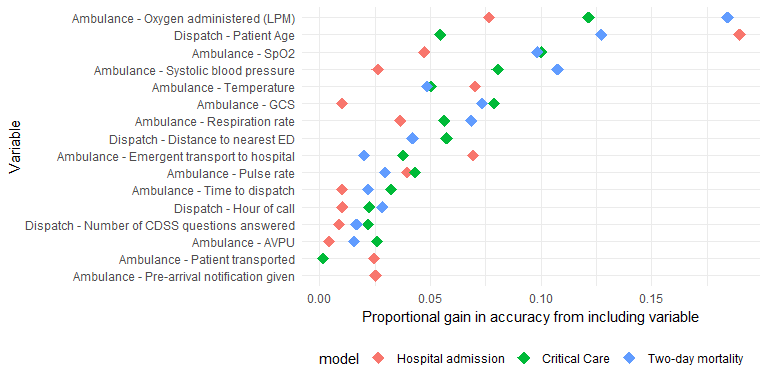
Figure 2
