# Supplementary material for: A validation of machine learning-based risk scores in the prehospital setting
Source: PLoS One. 2019 Dec 13;14(12):e0226518. doi: 10.1371/journal.pone.0226518 (PMC6910679; doi:10.1371/journal.pone.0226518)

## S1 Figure - Model calibration curves

### Overall


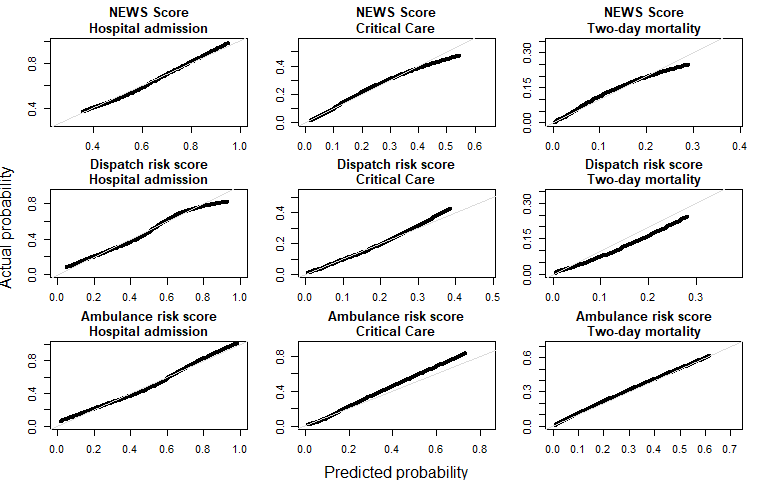


## Sub-group calibration

### By Age quartiles


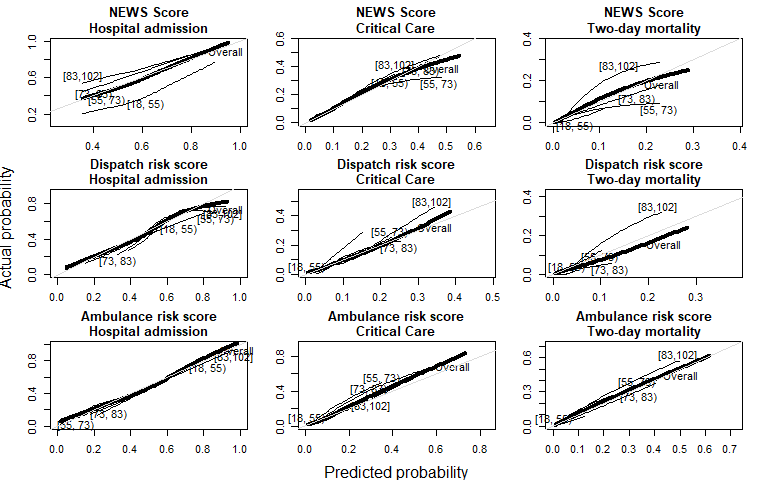


### By Gender


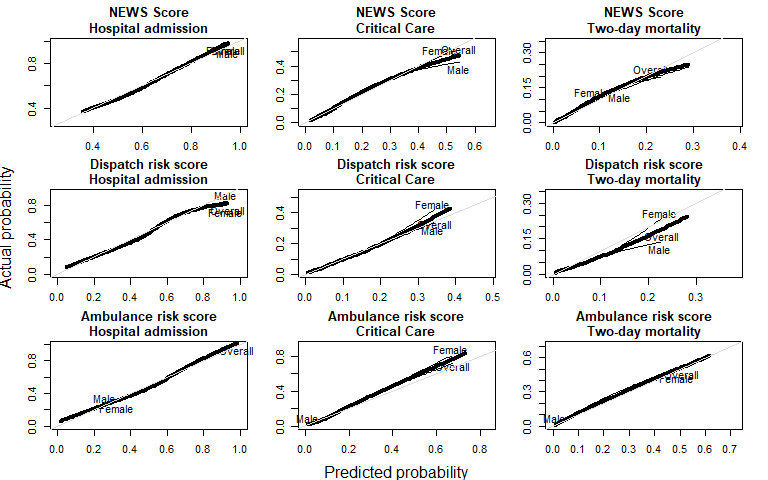


### By Priority


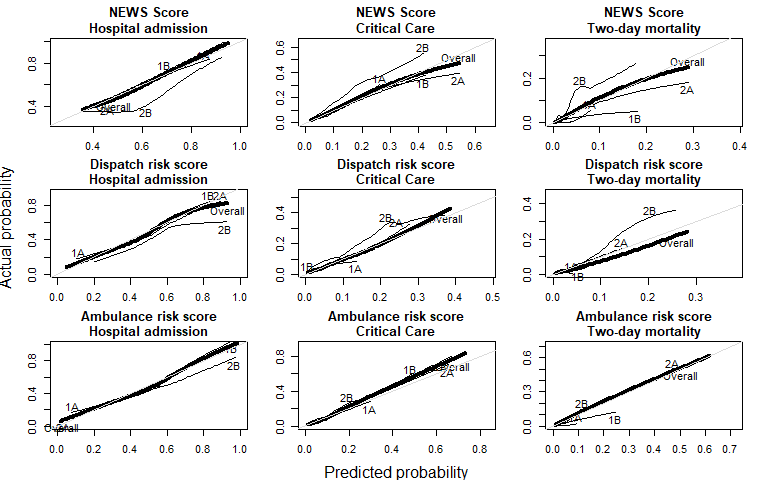


### By Common call types


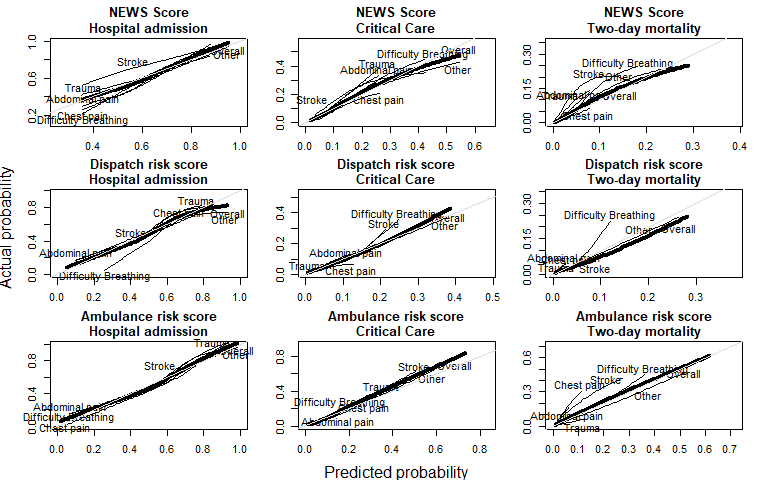

Supplement: S1 Fig — Provides the results of model calibration analyses using lowess smoothed calibration curves for both overall calibration, and calibration among sub-populations divided by age quartile, gender, call priority, and the 5 most common call types. (DOCX) [file pone.0226518.s003.docx]
