# Supplementary material for: A validation of machine learning-based risk scores in the prehospital setting
Source: PLoS One. 2019 Dec 13;14(12):e0226518. doi: 10.1371/journal.pone.0226518 (PMC6910679; doi:10.1371/journal.pone.0226518)
Supplement: S1 Table — Descriptions of each set of predictors included in gradient boosting models, providing information regarding the number of non-missing, non-zero values among included calls, the average gain provided by the predictor, and the number of dummy-encoded variables included from the predictor in the models. (DOCX) [file pone.0226518.s004.docx]

# S1 Table. Predictor description.

| Feature | Number of included calls with non-zero/non-missing value | Percent of included calls with non-zero/non-missing value | Average gain from inclusion of variables in ambulance models | Number of variables from group included in any ambulance model |
| --- | --- | --- | --- | --- |
| Ambulance - Airway findings | 33,952 | 88.9 | 0.095 | 3 |
| Ambulance - Any intervention provided | 29,529 | 77.3 | 0.131 | 1 |
| Ambulance - Breathing findings | 31,503 | 82.5 | 0.167 | 6 |
| Ambulance - Breathing sounds | 31,482 | 82.4 | 0.147 | 3 |
| Ambulance - Call types | 31,239 | 81.8 | 0.723 | 22 |
| Ambulance - Circulation findings | 32,393 | 84.8 | 0.148 | 4 |
| Ambulance - Critical patient status | 3,267 | 8.6 | 0.291 | 1 |
| Ambulance - Pre-arrival notification given | 5,986 | 15.7 | 1.443 | 1 |
| Ambulance - CPR administered | 55 | 0.1 | 0.243 | 1 |
| Ambulance - 12-lead EKG taken/sent to CICU | 9,128 | 23.9 | 0.397 | 1 |
| Ambulance - Patient immobilized | 764 | 2.0 | 0.131 | 1 |
| Ambulance - IV placed | 24,449 | 64.0 | 0.333 | 1 |
| Ambulance - Oxygen administered (LPM) | 6,522 | 17.1 | 13.173 | 1 |
| Ambulance - Lights & siren to hospital | 5,140 | 13.5 | 4.168 | 1 |
| Ambulance - Patient medical history | 16,130 | 42.2 | 0.272 | 7 |
| Ambulance - Medications administered | 22,365 | 58.5 | 1.301 | 12 |
| Ambulance - Priority to scene | 38,203 | 100.0 | 0.222 | 1 |
| Ambulance - Patient medications | 8,317 | 21.8 | 0.433 | 13 |
| Ambulance - Pulse quality | 33,019 | 86.4 | 0.428 | 7 |
| Ambulance - Skin condition | 29,858 | 78.2 | 0.251 | 3 |
| Ambulance - Time to dispatch | 19,815 | 51.9 | 1.856 | 1 |
| Ambulance - Time on scene | 35,315 | 92.4 | 2.894 | 1 |
| Ambulance - Time to hospital | 35,624 | 93.2 | 2.739 | 1 |
| Ambulance - AVPU | 37,172 | 97.3 | 1.894 | 1 |
| Ambulance - Systolic blood pressure | 37,710 | 98.7 | 6.878 | 1 |
| Ambulance - Respiration rate | 36,567 | 95.7 | 5.244 | 1 |
| Ambulance - GCS | 36,985 | 96.8 | 5.654 | 1 |
| Ambulance - Pulse rate | 37,755 | 98.8 | 3.624 | 1 |
| Ambulance - SpO2 | 38,028 | 99.5 | 9.342 | 1 |
| Ambulance - Temperature | 32,473 | 85.0 | 5.360 | 1 |
| Dispatch - Patient Age | 38,203 | 100.0 | 12.598 | 1 |
| Dispatch - CDSS category | 38,203 | 100.0 | 4.446 | 29 |
| Dispatch - Distance to nearest ED | 38,141 | 99.8 | 3.119 | 1 |
| Dispatch - Patient Gender | 19,814 | 51.9 | 0.257 | 1 |
| Dispatch - Hour of call | 37,070 | 97.0 | 1.654 | 1 |
| Dispatch - Hours since last contact | 38,203 | 100.0 | 0.478 | 1 |
| Dispatch - Number of prior contacts (30 days) | 4,906 | 12.8 | 0.143 | 1 |
| Dispatch - Month of call | 38,203 | 100.0 | 0.674 | 1 |
| Dispatch - Number of CDSS questions answered | 37,374 | 97.8 | 1.216 | 1 |
| Dispatch - CDSS questions | 37,151 | 97.2 | 5.035 | 144 |
| Dispatch - CDSS recommended priority | 38,203 | 100.0 | 0.396 | 1 |
