# Supplementary material for: A validation of machine learning-based risk scores in the prehospital setting
Source: PLoS One. 2019 Dec 13;14(12):e0226518. doi: 10.1371/journal.pone.0226518 (PMC6910679; doi:10.1371/journal.pone.0226518)
Supplement: S2 Table — Provides summary statistics in the form of the mean average calibration error for NEWS and ML risk scores both in the full population, and the weighted average of all investigated sub-populations. (DOCX) [file pone.0226518.s005.docx]

# S2 Table. Model calibration mean average error

| Stratification variable | Predictor | Mean absolute error from ideal calibration | | |
| --- | --- | --- | --- | --- |
|  |  | Hospital admission | Critical Care | Two-day mortality |
| Overall | NEWS Score | 0.0124 | 0.0027 | 0.0014 |
|  | Dispatch risk score | 0.0191 | 0.0030 | 0.0019 |
|  | Ambulance risk score | 0.0229 | 0.0071 | 0.0012 |
| Age | NEWS Score | 0.0564 | 0.0061 | 0.0032 |
|  | Dispatch risk score | 0.0257 | 0.0069 | 0.0028 |
|  | Ambulance risk score | 0.0264 | 0.0097 | 0.0020 |
| Gender | NEWS Score | 0.0126 | 0.0055 | 0.0017 |
|  | Dispatch risk score | 0.0191 | 0.0049 | 0.0021 |
|  | Ambulance risk score | 0.0230 | 0.0082 | 0.0016 |
| Priority | NEWS Score | 0.0179 | 0.0043 | 0.0029 |
|  | Dispatch risk score | 0.0230 | 0.0056 | 0.0030 |
|  | Ambulance risk score | 0.0254 | 0.0075 | 0.0021 |
| Call type | NEWS Score | 0.0356 | 0.0061 | 0.0032 |
|  | Dispatch risk score | 0.0262 | 0.0056 | 0.0025 |
|  | Ambulance risk score | 0.0264 | 0.0078 | 0.0029 |
