# Supplementary material for: A validation of machine learning-based risk scores in the prehospital setting
Source: PLoS One. 2019 Dec 13;14(12):e0226518. doi: 10.1371/journal.pone.0226518 (PMC6910679; doi:10.1371/journal.pone.0226518)
Supplement: S3 Table — Reports c-indexes for risk scores across a range of alternate weighting schemes, including the performance of individual model predictions across all investigated outcomes. (DOCX) [file pone.0226518.s006.docx]

# S3 Table - Sensitivity to alternate weights

| Predictor set | Weights* | Hospital admission | Critical Care | Two-day mortality |
| --- | --- | --- | --- | --- |
| Dispatch | 100:10:1 | 0.74 (0.73-0.75) | 0.68 (0.66-0.69) | 0.74 (0.71-0.78) |
|  | 4:2:1 | 0.73 (0.73-0.74) | 0.70 (0.68-0.72) | 0.78 (0.74-0.81) |
|  | 1:1:1 | 0.73 (0.72-0.73) | 0.70 (0.68-0.72) | 0.79 (0.76-0.82) |
|  | 1:2:4 | 0.71 (0.70-0.72) | 0.70 (0.68-0.72) | 0.79 (0.76-0.83) |
|  | 1:10:100 | 0.70 (0.70-0.71) | 0.68 (0.66-0.70) | 0.79 (0.76-0.83) |
|  | 1:0:0 | 0.74 (0.73-0.75) | 0.66 (0.64-0.68) | 0.72 (0.68-0.75) |
|  | 0:1:0 | 0.68 (0.68-0.69) | 0.71 (0.70-0.73) | 0.77 (0.74-0.81) |
|  | 0:0:1 | 0.68 (0.67-0.69) | 0.66 (0.64-0.68) | 0.79 (0.75-0.82) |
| Ambulance | 100:10:1 | 0.79 (0.79-0.80) | 0.76 (0.75-0.78) | 0.87 (0.84-0.89) |
|  | 4:2:1 | 0.79 (0.78-0.80) | 0.78 (0.76-0.80) | 0.89 (0.86-0.91) |
|  | 1:1:1 | 0.79 (0.78-0.80) | 0.79 (0.77-0.81) | 0.89 (0.87-0.92) |
|  | 1:2:4 | 0.78 (0.77-0.79) | 0.79 (0.78-0.81) | 0.90 (0.87-0.92) |
|  | 1:10:100 | 0.78 (0.77-0.78) | 0.79 (0.77-0.80) | 0.90 (0.87-0.92) |
|  | 1:0:0 | 0.79 (0.79-0.80) | 0.75 (0.73-0.77) | 0.83 (0.81-0.86) |
|  | 0:1:0 | 0.73 (0.72-0.74) | 0.80 (0.79-0.82) | 0.89 (0.87-0.92) |
|  | 0:0:1 | 0.72 (0.71-0.73) | 0.76 (0.74-0.78) | 0.89 (0.86-0.92) |

* Weights applied to model predictions for
Hospital Admission : Critical Care : Two-day Mortality
