# Supplementary material for: A validation of machine learning-based risk scores in the prehospital setting
Source: PLoS One. 2019 Dec 13;14(12):e0226518. doi: 10.1371/journal.pone.0226518 (PMC6910679; doi:10.1371/journal.pone.0226518)
Supplement: S4 Table — Presents patient volumes, ages, and outcomes across ED triage categories with more than 300 occurrences. (DOCX) [file pone.0226518.s007.docx]

## S4 Table. Descriptive statistics by ED diagnosis.

| ED triage category | N | Age, mean | Emergent transport, percent | NEWS value, mean | Critical care, percent | Admitted, percent | 2-day mortality, percent |
| --- | --- | --- | --- | --- | --- | --- | --- |
| Chest pain | 5054 | 67.3 (66.9-67.8) | 11.6 (10.7-12.4) | 2.34 (2.26-2.41) | 3.4 (2.9-3.9) | 40.1 (38.8-41.4) | 0.7 (0.5-0.9) |
| Difficulty Breathing | 4351 | 74.8 (74.4-75.3) | 19.5 (18.3-20.7) | 6.89 (6.77-6.99) | 10.9 (10.0-11.9) | 71.8 (70.4-73.1) | 2.4 (2.0-2.9) |
| Abdominal pain | 3716 | 57.0 (56.4-57.8) | 7.3 (6.4-8.1) | 2.24 (2.16-2.33) | 3.4 (2.8-4.0) | 42.1 (40.4-43.7) | 0.9 (0.6-1.2) |
| General weakness | 2431 | 77.0 (76.5-77.5) | 6.1 (5.2-7.0) | 3.82 (3.68-3.96) | 10.0 (8.8-11.3) | 70.7 (68.9-72.5) | 1.9 (1.4-2.4) |
| Neurological difficulty | 2321 | 72.4 (71.8-73.0) | 38.0 (36.1-39.9) | 2.45 (2.33-2.55) | 5.9 (4.9-6.9) | 73.9 (72.0-75.6) | 1.5 (1.0-1.9) |
| Hip injury | 1736 | 81.0 (80.5-81.6) | 1.0 (0.6-1.5) | 2.03 (1.93-2.14) | 5.0 (4.0-6.0) | 79.8 (77.9-81.7) | 0.7 (0.3-1.1) |
| Fever | 1571 | 74.3 (73.5-75.0) | 20.8 (18.9-22.9) | 7.47 (7.26-7.66) | 10.3 (8.8-11.9) | 86.4 (84.7-88.1) | 2.0 (1.4-2.7) |
| Dizziness | 1552 | 67.9 (67.0-68.9) | 1.1 (0.6-1.7) | 1.54 (1.45-1.63) | 0.8 (0.4-1.4) | 38.9 (36.5-41.4) | 0.3 (0.1-0.5) |
| Fainting | 1402 | 66.4 (65.3-67.4) | 6.3 (5.2-7.6) | 2.64 (2.50-2.79) | 2.4 (1.6-3.4) | 40.2 (37.7-42.7) | 0.5 (0.1-0.9) |
| Poisoning | 1225 | 39.0 (38.0-40.0) | 24.3 (21.9-26.8) | 3.86 (3.69-4.05) | 11.9 (10.2-13.8) | 51.5 (48.7-54.2) | 0.2 (0.0-0.4) |
| Cramping | 1147 | 49.4 (48.2-50.7) | 21.5 (19.2-23.8) | 4.44 (4.25-4.64) | 5.5 (4.2-6.9) | 43.8 (40.8-46.5) | 0.4 (0.1-0.9) |
| Head injury | 1128 | 68.6 (67.4-69.8) | 6.4 (5.0-7.9) | 2.06 (1.94-2.20) | 3.4 (2.2-4.3) | 22.8 (20.4-25.2) | 0.4 (0.1-0.7) |
| Arrythmia | 916 | 69.2 (68.1-70.2) | 13.3 (11.1-15.6) | 3.34 (3.17-3.52) | 2.6 (1.6-3.7) | 46.3 (43.1-49.7) | 0.5 (0.1-1.1) |
| Back pain | 721 | 58.5 (57.0-60.1) | 0.8 (0.3-1.7) | 1.36 (1.24-1.49) | 1.1 (0.4-2.1) | 31.3 (27.7-34.7) | 0.0 (0.0-0.0) |
| Major trauma | 681 | 55.0 (53.4-56.7) | 25.8 (22.6-29.2) | 2.35 (2.15-2.52) | 4.8 (3.2-6.6) | 28.9 (25.4-32.2) | 0.9 (0.3-1.6) |
| Reduced consciousness | 567 | 70.3 (68.6-71.9) | 60.3 (55.9-64.7) | 7.64 (7.32-7.98) | 25.0 (21.2-28.7) | 73.2 (69.7-76.7) | 10.2 (7.8-12.9) |
| Abdominal bleed | 509 | 72.8 (71.3-74.2) | 18.9 (15.7-22.2) | 3.41 (3.11-3.69) | 7.7 (5.5-10.0) | 74.7 (70.9-78.6) | 2.2 (1.0-3.5) |
| Minor trauma | 470 | 64.3 (62.1-66.6) | 4.9 (3.2-7.0) | 1.65 (1.49-1.81) | 0.6 (0.0-1.5) | 14.5 (11.1-17.7) | 0.0 (0.0-0.0) |
| Headache | 461 | 51.9 (50.2-53.7) | 8.7 (6.1-11.5) | 1.60 (1.43-1.79) | 2.2 (0.9-3.7) | 24.1 (20.4-28.2) | 0.9 (0.2-2.0) |
| Allergic Reaction | 429 | 45.3 (43.4-47.1) | 19.3 (15.6-23.1) | 2.55 (2.28-2.80) | 3.5 (1.9-5.4) | 10.0 (7.2-13.0) | 0.0 (0.0-0.0) |
| Shoulder injury | 404 | 59.6 (57.5-61.9) | 1.5 (0.5-2.7) | 1.57 (1.40-1.76) | 1.2 (0.2-2.5) | 24.5 (20.3-28.5) | 0.0 (0.0-0.0) |
| Foot/ankle injury | 331 | 52.2 (50.0-54.4) | 4.5 (2.4-6.9) | 1.44 (1.27-1.62) | 0.6 (0.0-1.5) | 44.4 (39.3-49.8) | 0.0 (0.0-0.0) |
| Swelling/pain in extremity | 322 | 72.1 (70.1-73.9) | 5.0 (2.8-7.5) | 2.29 (2.03-2.57) | 3.1 (1.2-5.3) | 50.9 (45.3-56.5) | 0.9 (0.0-2.2) |
| No ED Triage | 308 | 35.2 (33.6-37.0) | 9.7 (6.5-13.0) | 1.76 (1.56-1.98) | 0.0 (0.0-0.0) | 40.3 (34.7-45.8) | 0.0 (0.0-0.0) |
