# Supplementary material for: A validation of machine learning-based risk scores in the prehospital setting
Source: PLoS One. 2019 Dec 13;14(12):e0226518. doi: 10.1371/journal.pone.0226518 (PMC6910679; doi:10.1371/journal.pone.0226518)
Supplement: S1 Code — Provides all R code necessary to replicate the results reported in this manuscript in a user-provided dataset. If no dataset is provided, results are calculated in a randomly generated synthetic dataset mimicking the univariate properties of our data. This repository also includes the public release models and the code for an interactive demonstration of the models using the Shiny wed app framework. A maintained version of this code may be found on github: https://github.com/dnspangler/openTriage_validation, and an interactive demo app based on the public release models may be found here: https://ucpr.se/openTriage_demo/. (ZIP) [file pone.0226518.s008.zip › analysis.pdf]

A validation of machine learning-based risk scores in the prehospital setting

Douglas Spangler

Thomas Hermansson

David Smekal

Hans Blomberg

Table 1

	Training dataset (2016-2017)			Test dataset (2018)		
	Excluded, N	Excluded, percent	Remaining, N	Excluded, N	Excluded, percent	Remaining, N
Original			45045			23623
No dispatch CDSS data	2358	5.5	42687	857	3.8	22766
Missing PIN	2113	5.2	40574	1244	5.8	21522
No ambulance journal	2526	6.6	38048	933	4.5	20589
No ambulance transport	3879	11.4	34169	2461	13.6	18128
No hospital journal	3958	13.1	30211	1429	8.6	16699
No ED visit	2939	10.8	27272	1590	10.5	15109
Missing > 2 vitals	1336	5.2	25936	829	5.8	14280
Patient age < 18	1328	5.4	24608	685	5	13595
Included in study	20437	45.4	24608	10028	42.5	13595

Table 2

	Priority				
	1A	1B	2A	2B	Total
N	1283	15533	17227	4160	38203
Age, mean	56.2 (54.8-57.5)	64.5 (64.1-64.8)	67.5 (67.2-67.8)	67.3 (66.7-68.0)	65.9 (65.7-66.1)
Female, percent	46.1 (43.3-48.8)	49.4 (48.6-50.1)	53.9 (53.1-54.6)	54.5 (53.0-56.2)	51.9 (51.3-52.4)
Emergent transport , percent	38.7 (36.0-41.5)	24.6 (23.9-25.2)	4.3 (4.0-4.6)	2.2 (1.8-2.6)	13.5 (13.1-13.8)
Ambulance intervention*, percent	87.9 (86.0-89.8)	87.4 (86.8-87.9)	71.1 (70.4-71.8)	62.1 (60.6-63.5)	77.3 (76.9-77.7)
Missing vitals, percent	33.8 (30.9-36.5)	25.7 (25.0-26.4)	24.4 (23.8-25.0)	23.8 (22.5-25.0)	25.2 (24.7-25.6)
NEWS value, mean	5.79 (5.58-6.02)	3.76 (3.71-3.82)	2.97 (2.92-3.02)	2.40 (2.31-2.48)	3.32 (3.29-3.36)
Prior contacts (30 days), mean	0.21 (0.17-0.24)	0.17 (0.16-0.18)	0.17 (0.16-0.18)	0.23 (0.21-0.25)	0.18 (0.17-0.18)
Intensive Care Unit, percent	10.0 (8.3-11.6)	3.5 (3.2-3.8)	1.7 (1.5-1.8)	1.6 (1.2-1.9)	2.7 (2.5-2.8)
In-hospital death, percent	8.7 (7.2-10.2)	4.0 (3.7-4.3)	3.7 (3.4-4.0)	3.9 (3.4-4.5)	4.0 (3.8-4.2)
Critical care, percent	15.7 (13.6-17.5)	6.6 (6.2-7.0)	4.7 (4.4-5.0)	4.4 (3.8-5.1)	5.8 (5.6-6.0)
Admitted, percent	51.9 (49.0-54.6)	52.3 (51.6-53.1)	52.3 (51.6-53.1)	49.2 (47.5-50.6)	52.0 (51.5-52.5)
2-day mortality, percent	4.8 (3.7-6.0)	1.6 (1.4-1.8)	0.7 (0.6-0.9)	0.7 (0.5-1.0)	1.2 (1.1-1.3)

Figure 1

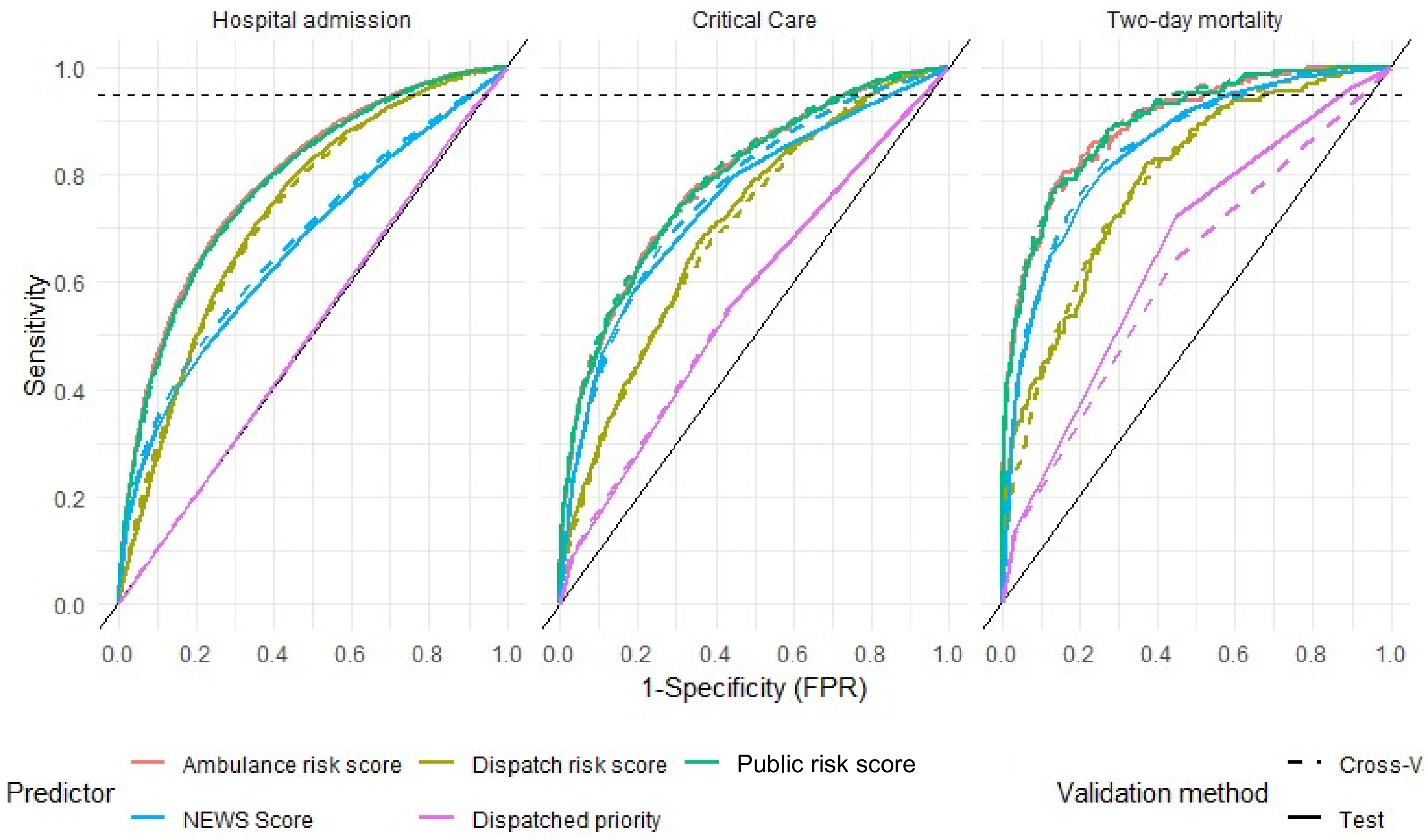

Hospital admission
Critical Care
Two-day mortality
Sensitivity
1.0
0.8
0.6
0.4
0.2
0.0
0.0 0.2 0.4 0.6 0.8 1.0
1-Specificity (FPR)
Predictor
Ambulance risk score
Dispatch risk score
Public risk score
NEWS Score
Dispatched priority
Validation method
Cross-V
Test

Table 3

Validation method	Outcome	Concordance index (95% CI) Dispatched priority	NEWS Score	Dispatch risk score	Ambulance risk score	Public risk score
Test	Hospital admission	0.51 (0.50-0.52)	0.66 (0.65-0.67)	0.73 (0.72-0.73)	0.79 (0.78-0.80)	0.78 (0.77-0.79)
	Critical Care	0.57 (0.55-0.59)	0.75 (0.73-0.77)	0.70 (0.68-0.72)	0.79 (0.77-0.81)	0.79 (0.77-0.80)
	Two-day mortality	0.66 (0.62-0.70)	0.85 (0.81-0.88)	0.79 (0.76-0.82)	0.89 (0.87-0.92)	0.89 (0.87-0.92)
Cross-Validated	Hospital admission	0.50 (0.50-0.51)	0.67 (0.67-0.68)	0.72 (0.72-0.73)	0.79 (0.78-0.79)	0.78 (0.78-0.79)
	Critical Care	0.57 (0.56-0.59)	0.76 (0.75-0.78)	0.70 (0.68-0.71)	0.79 (0.78-0.80)	0.79 (0.78-0.80)
	Two-day mortality	0.62 (0.58-0.65)	0.85 (0.83-0.87)	0.79 (0.77-0.81)	0.89 (0.87-0.91)	0.89 (0.88-0.91)

Table 4

Type	Priority	N	Emergent transport , percent	Ambulance intervention*, percent	NEWS value, mean	Critical care, percent	Admitted, percent	2-day mortality, percent
Current	1A	473	35.3 (31.3-39.3)	87.5 (84.6-90.5)	5.64 (5.26-6.00)	14.6 (11.6-18.2)	48.6 (44.4-53.5)	4.7 (2.8-6.8)
	1B	5657	22.5 (21.4-23.6)	86.4 (85.5-87.3)	3.70 (3.60-3.80)	6.5 (5.9-7.2)	52.3 (50.9-53.6)	1.7 (1.4-2.0)
	2A	6112	3.4 (3.0-3.9)	70.5 (69.4-71.7)	2.81 (2.74-2.89)	4.7 (4.1-5.2)	51.5 (50.3-52.8)	0.6 (0.4-0.9)
	2B	1353	1.7 (1.0-2.4)	60.5 (57.9-63.3)	2.33 (2.19-2.46)	4.4 (3.4-5.4)	47.7 (45.0-50.3)	0.4 (0.1-0.8)
Hypo-thetical	1A	473	37.8 (33.4-42.1)	90.7 (87.9-93.2)	7.22 (6.85-7.61)	22.2 (18.4-26.0)	75.3 (71.0-79.3)	10.1 (7.6-13.1)
	1B	5657	15.3 (14.4-16.2)	77.9 (76.9-78.9)	4.22 (4.12-4.32)	8.2 (7.5-8.9)	69.6 (68.5-71.0)	1.6 (1.2-1.9)
	2A	6112	8.9 (8.2-9.7)	74.9 (73.8-76.0)	2.37 (2.31-2.44)	3.3 (2.8-3.7)	40.3 (39.1-41.5)	0.4 (0.3-0.6)
	2B	1353	6.1 (4.9-7.3)	75.0 (72.8-77.3)	1.55 (1.46-1.64)	1.0 (0.5-1.6)	16.3 (14.3-18.3)	0.0 (0.0-0.0)

Figure 2

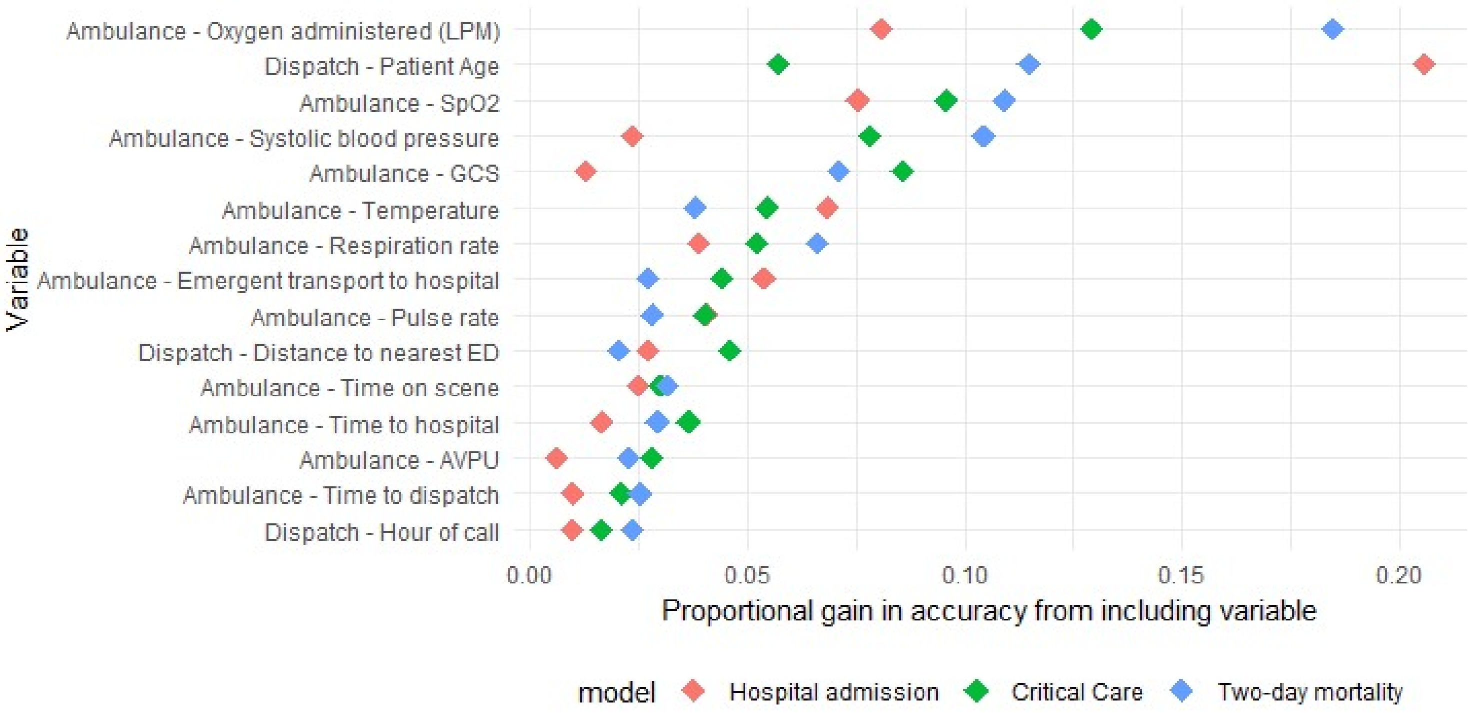
Ambulance - Oxygen administered (LPM)
Dispatch - Patient Age
Ambulance - SpO2
Ambulance - Systolic blood pressure
Ambulance - GCS
Ambulance - Temperature
Ambulance - Respiration rate
Ambulance - Emergent transport to hospital
Ambulance - Pulse rate
Dispatch - Distance to nearest ED
Ambulance - Time on scene
Ambulance - Time to hospital
Ambulance - AVPU
Ambulance - Time to dispatch
Dispatch - Hour of call
Variable
0.00
0.05
0.10
0.15
0.20
Proportional gain in accuracy from including variable
model
Hospital admission
Critical Care
Two-day mortality
