# Supplementary figures and images for: A validation of machine learning-based risk scores in the prehospital setting
Source: PLoS One. 2019 Dec 13;14(12):e0226518. doi: 10.1371/journal.pone.0226518 (PMC6910679; doi:10.1371/journal.pone.0226518)

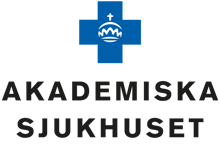

Supplement: S1 Code — Provides all R code necessary to replicate the results reported in this manuscript in a user-provided dataset. If no dataset is provided, results are calculated in a randomly generated synthetic dataset mimicking the univariate properties of our data. This repository also includes the public release models and the code for an interactive demonstration of the models using the Shiny wed app framework. A maintained version of this code may be found on github: https://github.com/dnspangler/openTriage_validation, and an interactive demo app based on the public release models may be found here: https://ucpr.se/openTriage_demo/. (ZIP) [file pone.0226518.s008.zip › demo/www/as.png]

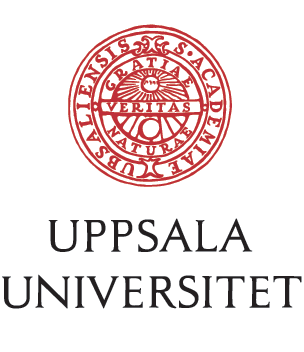

Supplement: S1 Code — Provides all R code necessary to replicate the results reported in this manuscript in a user-provided dataset. If no dataset is provided, results are calculated in a randomly generated synthetic dataset mimicking the univariate properties of our data. This repository also includes the public release models and the code for an interactive demonstration of the models using the Shiny wed app framework. A maintained version of this code may be found on github: https://github.com/dnspangler/openTriage_validation, and an interactive demo app based on the public release models may be found here: https://ucpr.se/openTriage_demo/. (ZIP) [file pone.0226518.s008.zip › demo/www/uu.png]

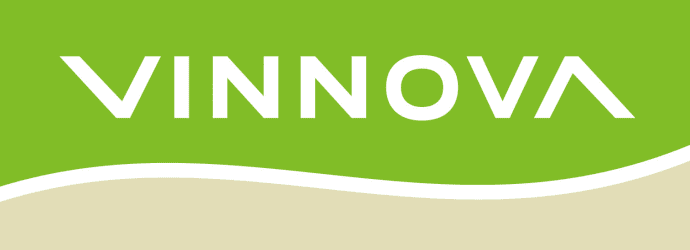

Supplement: S1 Code — Provides all R code necessary to replicate the results reported in this manuscript in a user-provided dataset. If no dataset is provided, results are calculated in a randomly generated synthetic dataset mimicking the univariate properties of our data. This repository also includes the public release models and the code for an interactive demonstration of the models using the Shiny wed app framework. A maintained version of this code may be found on github: https://github.com/dnspangler/openTriage_validation, and an interactive demo app based on the public release models may be found here: https://ucpr.se/openTriage_demo/. (ZIP) [file pone.0226518.s008.zip › demo/www/vinnova.png]
